# Supplementary material for: Synergistic Activity and Mechanism of Sanguinarine with Polymyxin B against Gram-Negative Bacterial Infections
Source: Pharmaceutics. 2024 Jan 3;16(1):70. doi: 10.3390/pharmaceutics16010070 (PMC10820148; doi:10.3390/pharmaceutics16010070)
Supplement: Supplementary file 1 [file pharmaceutics-16-00070-s001.zip › Supplementary Information.pdf]

Supplementary Information

Figure S1. *In vivo* antibacterial activity of PMB-SA combination in murine peritonitis model. **a** The fold changes of white pulp area in spleens. **b** Leukocyte and neutrophil count in blood (n = 5) **c** Assays of proinflammatory cytokines IL-6, IL-1β and TNF-α in livers, kidneys and spleens by RT-qPCR (n = 5). All data were expressed as mean ± SD (error bars). **P* < 0.05, ***P* < 0.01, ****P* < 0.001, *****P* < 0.0001. The survival rate was analyzed by a log-rank (Mantel-Cox) test. Welch's one-way ANOVA with Dunnett's T3 multiple comparison test were used for analysis.

Figure S2. Dot-plot profiles of the bacterial membrane permeability assay. The Ctrl-v group represented the viable control with untreated bacteria. The Ctrl-d group represented the dead control with bacteria killed by heating. The quadrant I and II were designated based on the dot-plot profiles of Ctrl-v and Ctrl-d respectively.

Figure S3. Metabolomic analysis of *E. coli* treated with PMB and SA for 1h. **a** Principal component analysis (PCA) plots for metabolite levels from *E. coli* ATCC 25922 significantly impacted by PMB (2 × MIC), SA (2 × MIC), and their combination. **b** Venn diagrams exhibiting the number of metabolites significantly affected in each group (*P* ≤ 0.05). **c** Changing numbers of significant metabolites in different groups, including lipids, nucleosides, amino acids, carbohydrates and so on. (*P* ≤ 0.05). **d** Integrated pathway map of metabolites in *E. coli* ATCC 25922 significantly impacted by PMB, SA, and their combination in pathways of oxidative phosphorylation and pantothenate and coenzyme A biosynthesis. Bar charts for the significantly impacted metabolites of these pathways following PMB (2 × MIC), SA (2 × MIC) or their combination at 1 h (*P* < 0.05). **e** Heat map profiles of relative abundance of significantly perturbed metabolites in DNA and RNA metabolism, including purine metabolism (hypoxanthine, adenine and guanine) and pyrimidine metabolism (uracil, thymine and cytosine) (P ≤ 0.05), **P* < 0.05, ***P* < 0.01, ****P* < 0.001, *****P* < 0.0001. All data were obtained from six replicates (n = 6), and were expressed as mean ± SD (error bars). Student's t-test was used to analyze.
