# Supplementary material for: Synergistic Activity and Mechanism of Sanguinarine with Polymyxin B against Gram-Negative Bacterial Infections
Source: Pharmaceutics. 2024 Jan 3;16(1):70. doi: 10.3390/pharmaceutics16010070 (PMC10820148; doi:10.3390/pharmaceutics16010070)
Supplement: Supplementary file 1 [file pharmaceutics-16-00070-s001.zip › Table S1.pdf]

Table S1 Sequence of primers used for RT-qPCR in *in vivo* study

**Gene**	**Forward primer (5′-3′)**	**Reverse primer (5′-3′)**
TNF-α	CACAGAAAGCATGATCCGCG	ACTGATGAGAGGGAGGCCAT
IL-1β	ACTCATTGTGGCTGTGGAGA	TTGTTCATCTCGGAGCCTGT
IL-6	CACTTCACAAGTCGGAGGCT	AATGAATGGACGCCCACACT
GAPDH	CTCCCACTCTTCCACCTTCG	TAGGGCCTCTCTTGCTCAGT
