# Supplementary figures and images for: Synergistic Activity and Mechanism of Sanguinarine with Polymyxin B against Gram-Negative Bacterial Infections
Source: Pharmaceutics. 2024 Jan 3;16(1):70. doi: 10.3390/pharmaceutics16010070 (PMC10820148; doi:10.3390/pharmaceutics16010070)

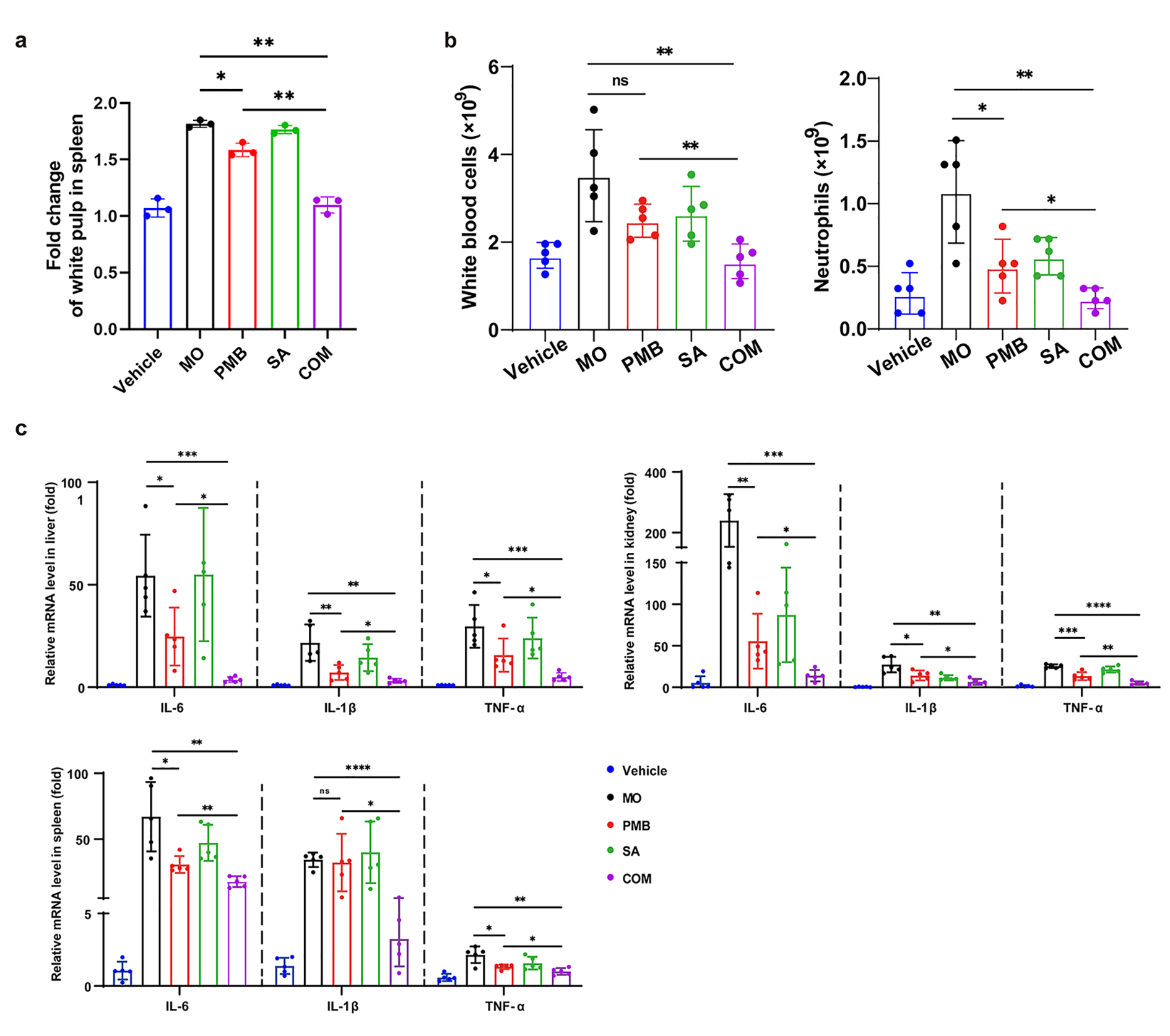

Supplement: Supplementary file 1 [file pharmaceutics-16-00070-s001.zip › Figure S1.tif]

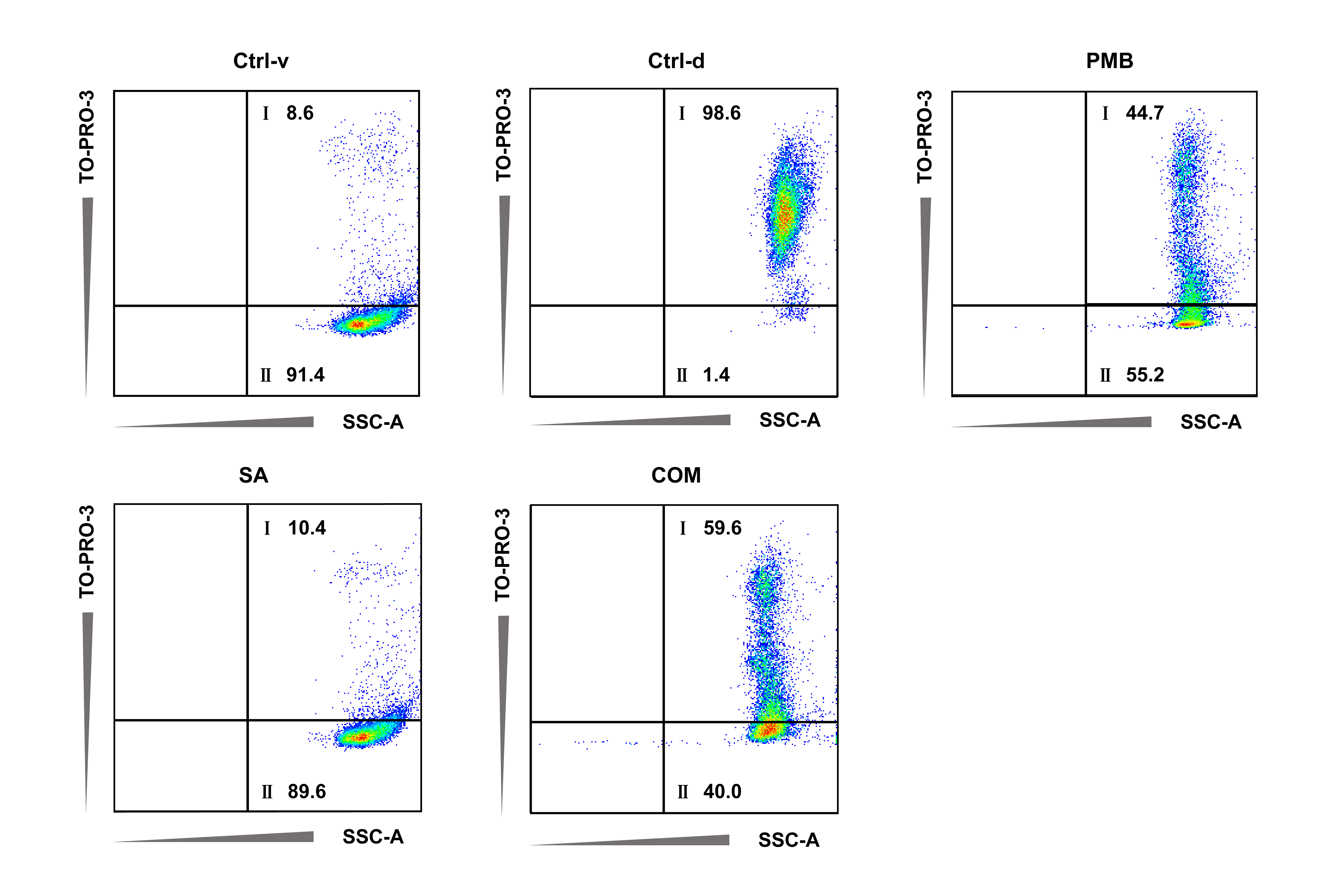

Supplement: Supplementary file 1 [file pharmaceutics-16-00070-s001.zip › Figure S2.tif]

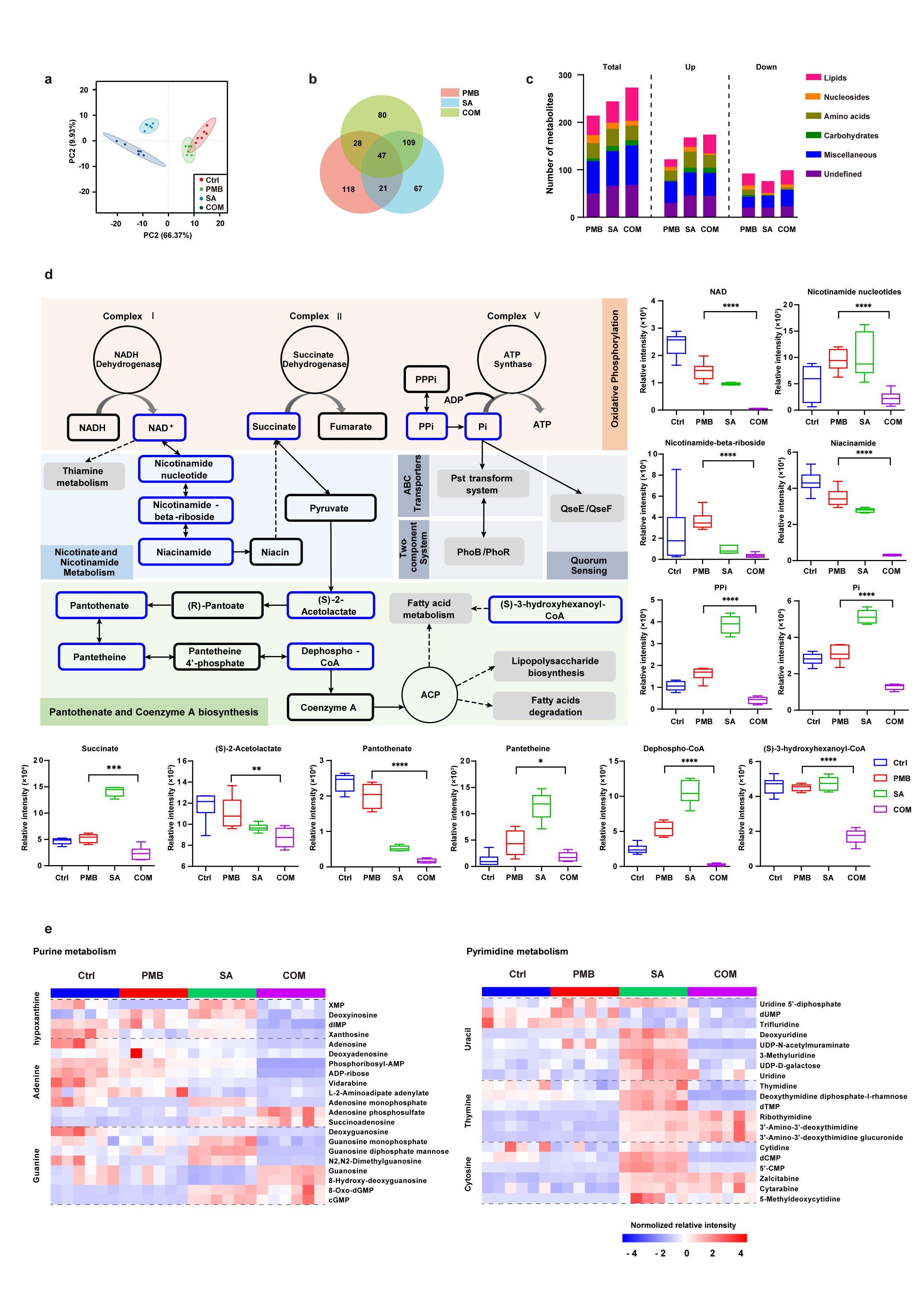

Supplement: Supplementary file 1 [file pharmaceutics-16-00070-s001.zip › Figure S3.tif]
